# Adjunctive topical mesenchymal stem cell-derived secretome-enriched biological dressings in pediatric complex wounds: a retrospective case series

**DOI:** 10.3389/fmed.2026.1795253

**Published:** 2026-05-19

**Authors:** José Ramón García-Lira, Adriana Maria Valencia-Herrera, Mirna Eréndira Toledo-Bahena, Carlos Alfredo Mena-Cedillos, Adrián Chávez-López

**Affiliations:** 1 Department of Pediatric Dermatology, Federico Gómez Children’s Hospital, Mexico City, Mexico; 2 Intensive Care Unit, Federico Gómez Children’s Hospital, Mexico City, Mexico

**Keywords:** adjunctive therapy, complex wound, extracellular vesicles, mesenchymal stem cells, pediatric dermatology, regenerative medicine, therapeutic strategies, wound healing

## Abstract

**Background:**

Mesenchymal stem cell (MSC)-derived secretome, including extracellular vesicles (EVs), has emerged as a potential cell-free regenerative approach due to its immunomodulatory and tissue repair-associated properties. While topical applications have been explored in adult dermatologic settings, clinical evidence in pediatric patients with complex cutaneous wounds remains scarce.

**Methods:**

A retrospective observational case series was conducted at a tertiary referral pediatric hospital. Medical records and photographic documentation of six pediatric patients with complex cutaneous wounds treated with topical biological dressings containing MSC-derived secretome enriched in EVs were reviewed between January 2020 and January 2024. All patients received standard-of-care management in addition to adjunctive application of the biological dressings. Clinical evolution and treatment tolerability were assessed descriptively.

**Results:**

Progressive re-epithelialization and clinical improvement were observed across all cases based on clinical and photographic assessment. Time to complete re-epithelialization ranged from 6 to 14 days across heterogeneous wound etiologies. Estimated wound reduction ranged from approximately 85 to >90% at defined time points. No treatment-related adverse events were documented during follow-up.

**Conclusion:**

In this retrospective case series, progressive re-epithelialization was documented during follow-up in pediatric patients with complex cutaneous wounds receiving adjunctive topical MSC-derived secretome-enriched biological dressings alongside standard care. However, given the observational design, small sample size, heterogeneous diagnoses, and absence of a control group, these findings should be interpreted as descriptive and hypothesis-generating. Prospective, controlled studies are needed to better define the therapeutic role and safety profile of these interventions in pediatric regenerative dermatology.

## Introduction

Skin integrity is essential for maintaining homeostasis and providing a protective barrier against mechanical, chemical, and microbial insults (1–4). In pediatric patients, disruption of this barrier due to trauma, burns, surgical procedures, or complex wounds represents a significant clinical challenge, as impaired healing may lead to infection, functional limitations, prolonged hospitalization, and long-term physical and psychological sequelae. Despite advances in wound care, conventional therapies may be insufficient to achieve optimal tissue regeneration in complex or refractory cases (4, 5).

Mesenchymal stem cells (MSCs) have emerged as a promising therapeutic approach in regenerative medicine due to their immunomodulatory, angiogenic, and reparative properties (6, 7). Increasing evidence has suggested that these effects are primarily mediated through paracrine mechanisms rather than direct cellular engraftment or differentiation (7, 8). Among these mediators, the MSC-derived secretome, which includes extracellular vesicles (EVs), soluble proteins, and bioactive molecules, has gained significant attention as a potential cell-free therapeutic strategy.

EVs, including exosomes, are nano-sized membrane-bound particles released by MSCs that contain proteins, lipids, and nucleic acids capable of modulating the wound microenvironment. Preclinical studies have demonstrated that MSC-derived secretome can promote angiogenesis, support keratinocyte and fibroblast activity, regulate inflammatory responses, and contribute to extracellular matrix remodeling, processes that are central to wound repair (8–12). These properties have generated interest in EV-enriched formulations as a potential adjunct in regenerative therapies.

Clinical applications in dermatology have primarily been explored in adult populations, particularly in procedural and esthetic settings, where EV-based or exosome-enriched products have been used as adjunctive treatments to enhance skin recovery. While these studies suggest potential benefits, the available evidence remains limited, heterogeneous, and predominantly focused on adult patients (8–12).

Different biological and clinical factors, including skin structure, immune response, and regenerative dynamics, must be considered while treating pediatric wound healing. In addition, therapeutic interventions in children must prioritize safety, tolerability, and minimal invasiveness. Currently, clinical data evaluating the topical use of MSC-derived secretome or EV-based therapies in pediatric patients with complex wounds are scarce.

This study aimed to describe the clinical evolution and safety profile of pediatric patients with complex cutaneous wounds treated with topical MSC-derived secretome-enriched biological dressings as an adjunct to standard care in a tertiary referral center. By reporting real-world clinical experience, this case series aimed to contribute preliminary data to a field with limited pediatric evidence and to inform future studies evaluating the role of EV-based therapies in regenerative dermatology.

## Methods

### Study design and setting

This study was a retrospective observational case series conducted at the Hospital Infantil de México Federico Gómez, National Institute of Health, a tertiary referral pediatric hospital in Mexico City. Medical records and photographic documentation were reviewed for pediatric patients treated with topical biological dressings containing MSC-derived secretome enriched in EVs between January 2020 and January 2024.

### Patient selection

Pediatric patients were eligible for inclusion if they met the following criteria: (1) age under 18 years at the time of treatment; (2) presence of complex cutaneous wounds, including inflammatory, autoimmune, or ulcerative lesions; and (3) treatment with topical MSC-derived secretome-enriched biological dressings as an adjunctive therapy. Patients with incomplete clinical records or insufficient follow-up documentation were excluded from the study.

### Description of the biological dressing

Topical biological dressings used in this study consisted of a standardized, commercially available product (ExomeSkin®, ExomeLab, Mexico City, México) derived from MSCs obtained from placental tissue.

MSC characterization provided by the manufacturer was conducted using flow cytometry and demonstrated a high expression of CD73 (99.98%), CD90 (99.95%), and CD105 (99.8%). Hematopoietic and immune markers, such as CD34, CD45, HLA-DR, CD14, CD11b, and CD19, were absent, which is consistent with the established criteria for mesenchymal stromal cells.

The final formulation consists of an MSC-derived secretome enriched in EVs embedded within a calcium alginate and medical-grade collagen matrix. The scaffold functions as a three-dimensional biological matrix designed to support wound coverage and facilitate the local delivery of bioactive factors. Additional manufacturer-provided characterization includes flow cytometry analysis demonstrating the presence of vesicle-associated markers, including CD9, CD63, and CD81, as well as lipid membrane-bound EVs. However, detailed particle quantification, size distribution, and purity metrics (e.g., nanoparticle tracking analysis) were not available.

Although the scaffold is designed to support local retention and gradual exposure of bioactive components, release kinetics and pharmacodynamic properties were not formally evaluated in this clinical setting.

The dressing was supplied as a topical biological scaffold and applied directly to the wound bed following routine wound preparation. The same formulation was used consistently across all patients.

### Treatment protocol

All patients received standard-of-care treatment according to the underlying pathology, including wound cleaning, debridement when indicated, infection control, and appropriate systemic or topical therapies. The MSC-derived secretome-enriched biological dressing was applied as adjunctive therapy.

Application frequency and duration were individualized based on wound characteristics and clinical response. Dosing was determined clinically according to wound surface area and anatomical location, as quantitative vesicle concentration data were not available.

Dressings were replaced every 72 h, and wound care included irrigation with sterile physiological saline every 8 h, according to institutional protocols and clinical assessment.

### Outcome measures and data collection

Demographic and clinical data were collected from electronic medical records, including age, sex, diagnosis, wound characteristics, anatomical location, and duration prior to treatment.

Clinical outcomes were assessed through medical records and serial photographic documentation. Wound size (% total body surface area, TBSA) and percentage reduction were retrospectively estimated based on serial clinical photographs and anatomical surface area approximation using standard regional distribution models. Based on anatomical landmarks, the approximate surface area of localized lesions was determined. Percentage wound reduction was estimated by comparing baseline and follow-up images, focusing on the extent of re-epithelialized versus denuded tissue. These assessments were performed by the treating clinical team and should therefore be interpreted as semi-quantitative.

The primary outcome was time to complete re-epithelialization, defined as full closure of the wound surface without exudate or need for further dressing. The secondary outcomes included estimated wound size reduction, clinical evolution, and treatment tolerability. Adverse events were recorded when documented in the clinical record.

### Ethics statement

The study was conducted in accordance with the principles of the Declaration of Helsinki. Approval was obtained from the Institutional Research and Ethics Committee of the Hospital Infantil de México Federico Gómez (HIM-2022-067). Written informed consent for treatment and the use of clinical data and images for scientific purposes was obtained from the patients’ parents or legal guardians.

## Results

### Baseline patient and wound characteristics

A total of six pediatric patients were included in this retrospective case series. Baseline demographic and clinical characteristics, including wound size, duration prior to treatment, and treatment parameters, are summarized in Table 1.

**Table 1:** Clinical characteristics, treatment course, and outcomes of pediatric patients treated with topical MSC-derived secretome-enriched biological dressings.

Case	Sex	Age	Diagnosis	Wound location	Wound size (%TBSA)	Duration prior to treatment^a^ (days)	Concomitant systemic therapy^b^	Dressings per application^c^ (*n*)	Number of applications (*n*)	Time to complete re-epithelialization (days)	Estimated wound reduction (%)	Follow-up
1	F	14 years	Toxic epidermal necrolysis (TEN)	Trunk and upper extremities	~20%	1	Supportive care + IVIG	30	3	14	>90% (estimated, days 10–14)	3 months
2	F	9 years	Toxic epidermal necrolysis (TEN)	Trunk and lower extremities	~22%	1	Supportive care + systemic corticosteroids	28	4	10	~85–90% (estimated, day 6)	3 months
3	F	16 years	Stevens–Johnson syndrome (SJS)	Oral mucosa and face	~2.5%	1	Supportive care + systemic corticosteroids	2	3	7	~90–95% (estimated, day 7)	3 months
4	F	8 years	Pemphigus foliaceus	Posterior trunk	~4%	1	Systemic steroids + azathioprine	2	2	6	~90% (estimated, day 6)	6 months
5	M	16 years	Discoid lupus erythematosus (DLE)	Scalp	~1.5%	2	Hydroxychloroquine + topical steroids	2	2	6	~85–90% (estimated, day 6)	5 months
6	M	3 months	Ulcerated infantile hemangioma	Central face	~0.5%	1	Propranolol + wound care	1	3	7	~90–95% (estimated, day 7)	6 months

a Time from clinical diagnosis or onset of significant skin detachment to the initiation of adjunctive treatment.

b Concomitant systemic therapy refers to standard-of-care systemic treatments administered for the underlying condition during the wound-healing period.

c Dressings per application refers to the number of individual MSC-derived secretome-enriched biological dressings applied during a single treatment session, adjusted according to wound surface area and anatomical location.

The cohort was heterogeneous and included immune-mediated conditions, such as toxic epidermal necrolysis (TEN), Stevens–Johnson syndrome (SJS), pemphigus foliaceus, and discoid lupus erythematosus, as well as a vascular lesion, such as ulcerated infantile hemangioma. The estimated wound size ranged from approximately 0.5 to 22% of the TBSA, and the duration prior to initiation of adjunctive treatment ranged from 1 to 3 days.

All patients received standard-of-care management appropriate to their underlying diagnosis in addition to adjunctive application of MSC-derived secretome-enriched biological dressings.

### Wound healing evolution

**Figure 1:**
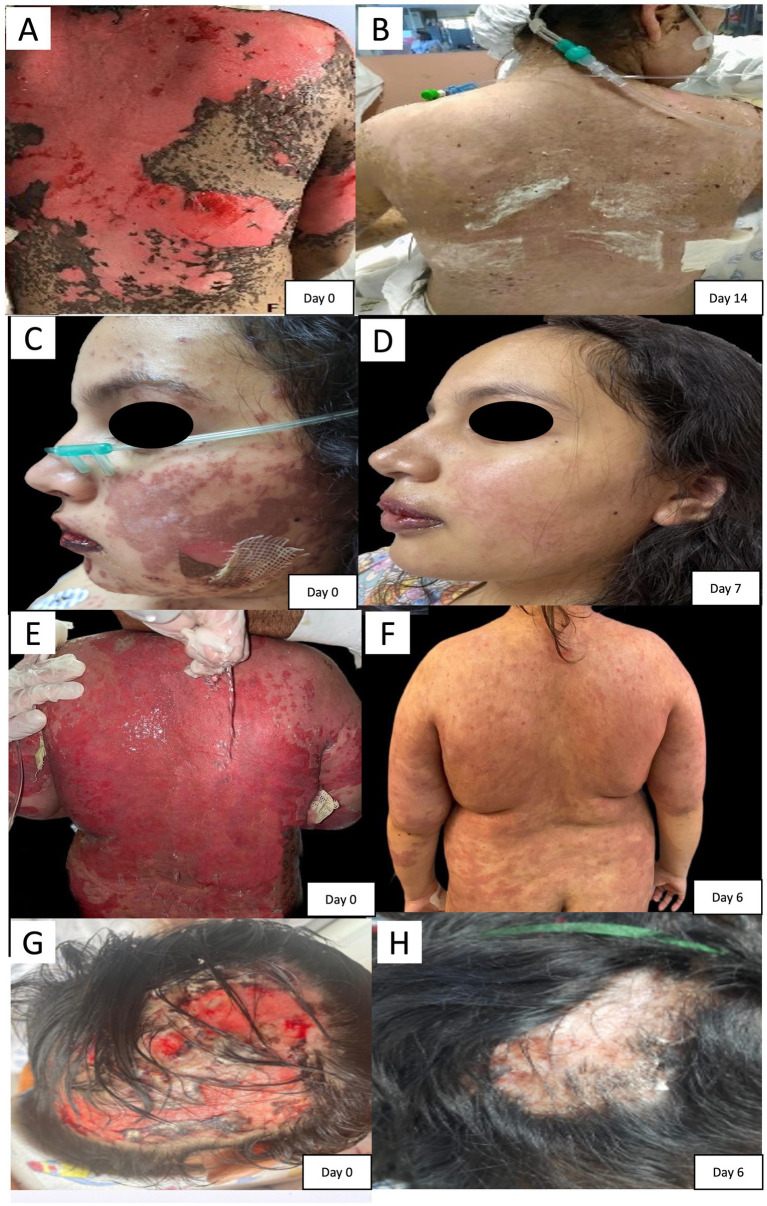
Representative clinical photographs showing the evolution of complex pediatric cutaneous wounds treated with topical MSC-derived secretome-enriched biological dressings as an adjunct to standard wound care. Panels **(A,C,E,G)** depict baseline lesions prior to initiation of adjunctive therapy. Panels **(B,D,F,H)** show the corresponding post-treatment appearance following clinical re-epithelialization. The interval between baseline and post-treatment ranged from 6 to 14 days, depending on the underlying diagnosis. No local complications or abnormal scarring were documented during available follow-up.

Progressive wound healing was observed across all cases based on clinical assessment and serial photographic documentation (Figure 1).

Given the small sample size, individual values are reported in Table 1 rather than relying solely on summary statistics. Time to complete re-epithelialization ranged from 6 to 14 days. The number of applications ranged from 2 to 4 per patient, while the number of dressings used per session ranged from 1 to 30, depending on wound extent and anatomical location.

Based on retrospective photographic assessment, the estimated wound reduction ranged from approximately 85 to >90% across cases at defined time points (Table 1).

**Figure 2:**
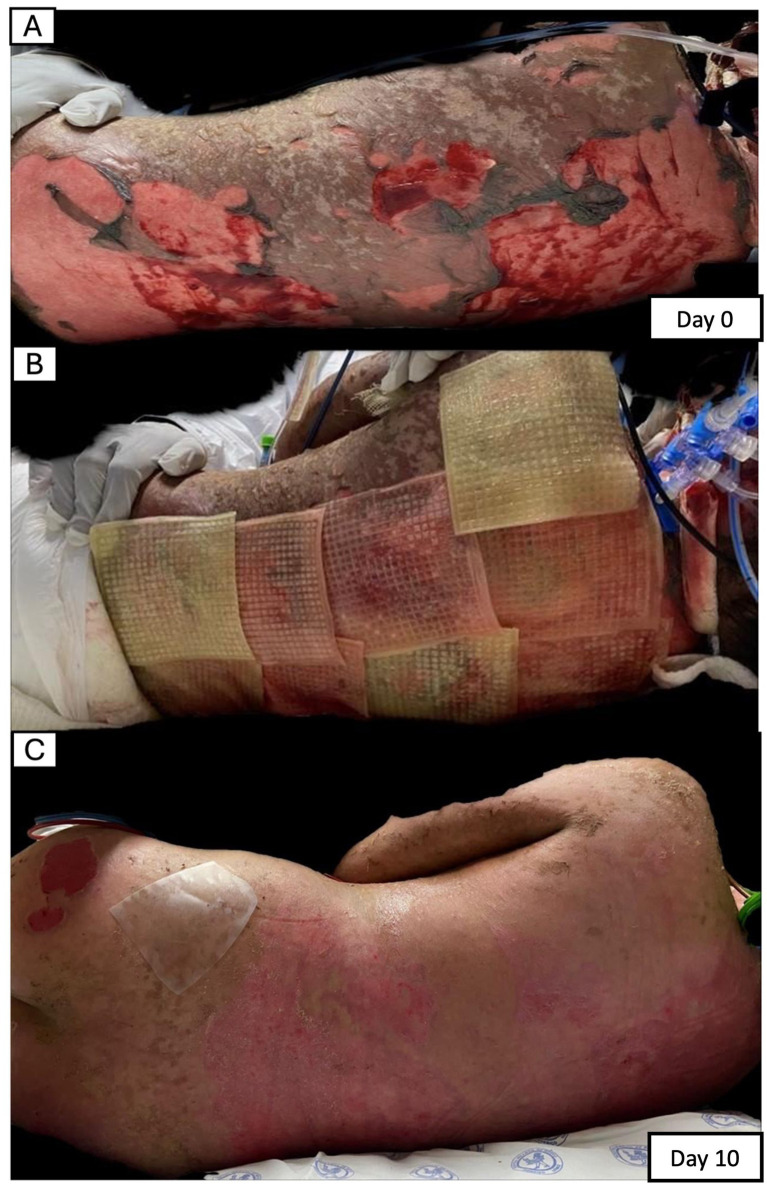
Sequential clinical photographs demonstrating wound evolution in a pediatric patient with TEN treated with adjunctive topical MSC-derived secretome-enriched biological dressings. **(A)** Extensive epidermal detachment prior to adjunctive therapy (day 0). **(B)** Application of biological dressings (day 0). **(C)** Clinical appearance at follow-up showing re-epithelialization (day 10). All images correspond to the same anatomical region. The patient received standard-of-care systemic therapy in addition to topical dressing application.

**Figure 3:**
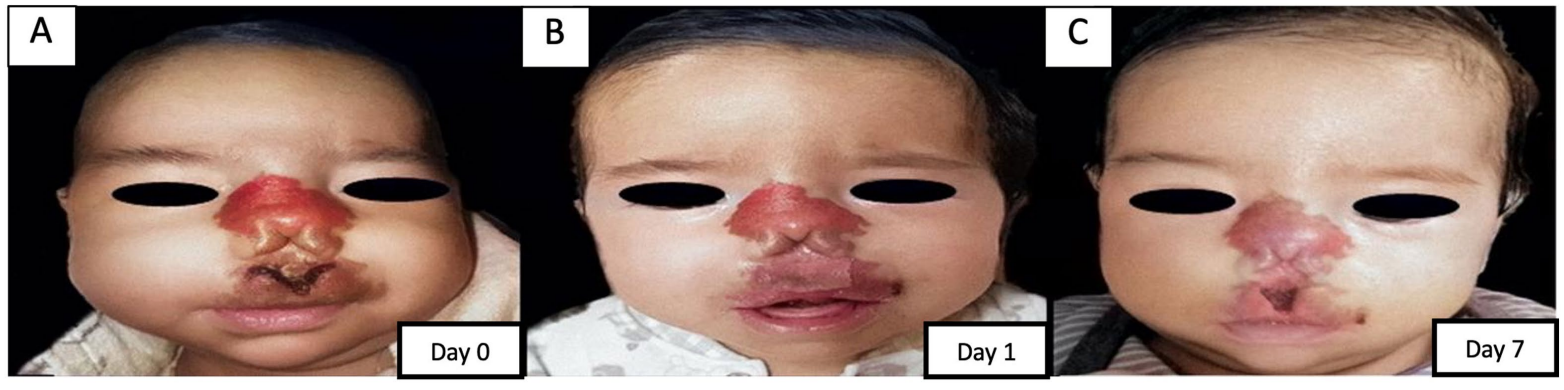
Sequential clinical photographs showing the evolution of an ulcerated infantile hemangioma treated with adjunctive topical MSC-derived secretome-enriched biological dressings. **(A)** Baseline ulcerated lesion with central necrotic tissue and fistula formation (day 0). **(B)** Early follow-up demonstrating a reduction in ulceration and progressive re-epithelialization and closure of the fistulous opening (day 1). **(C)** Follow-up image showing restoration of cutaneous integrity (day 7). The patient received systemic propranolol in addition to topical biological dressing application.

Figures 2, 3 present representative cases selected to illustrate the spectrum of wound etiologies included in the series, based on the availability of the most complete serial photographic documentation. Figure 2 presents the evolution of a patient with TEN, with extensive baseline epidermal detachment and subsequent re-epithelialization by day 10. Figure 3 shows the progression of an ulcerated infantile hemangioma with restoration of cutaneous integrity by day 7.

All cases received concomitant standard therapies, including systemic immunomodulatory or supportive treatments, and the biological dressings were used as an adjunctive intervention. Due to the heterogeneity of diagnoses and concurrent treatments, no causal relationship between the intervention and clinical outcomes can be established.

### Safety and tolerability

No treatment-related adverse events were documented during available follow-up.

## Discussion

### Principal findings

This retrospective case series describes the clinical course of pediatric patients with complex cutaneous wounds treated with MSC-derived secretome-enriched biological dressings as an adjunct to standard therapy. Across all cases, progressive re-epithelialization and documented clinical evolution were observed, with no documented adverse events during follow-up.

Given the observational design, small sample size, and absence of a control group, these findings do not establish a causal relationship between the intervention and the observed clinical outcomes. Rather, they reflect real-world clinical observations in a heterogeneous cohort receiving combined standard-of-care and adjunctive therapy.

The included cases encompassed a range of immune-mediated and vascular conditions, including TEN, SJS, autoimmune blistering disease, discoid lupus erythematosus, and ulcerated infantile hemangioma. These conditions differ substantially in pathophysiology, expected healing trajectories, and response to systemic therapy, limiting direct comparison across cases and precluding generalization of outcomes (13, 14).

In severe cutaneous adverse reactions such as TEN and SJS, re-epithelialization under optimized supportive care and systemic immunomodulatory therapy is typically reported within approximately 14–21 days (13). Similarly, ulcerated infantile hemangiomas treated with systemic *β*-blockers may require several weeks for complete healing. The time to re-epithelialization observed in this series (6–14 days) falls within the expected clinical range for these conditions. Accordingly, these findings should not be interpreted as evidence of accelerated healing, but rather as documentation of consistent and uncomplicated re-epithelialization in patients receiving adjunctive biological dressings. Although causality cannot be inferred, the observed clinical evolution is consistent with previously reported studies evaluating EV-based therapies in dermatologic settings. In adult populations, MSC-derived EVs have been associated with improved skin regeneration and recovery following procedural interventions when used as adjunctive treatments. However, direct comparison remains limited due to differences in patient populations, wound types, and study design (8–11).

### Biological and mechanistic considerations

From a biological perspective, MSC-derived secretome enriched in EVs contains a complex mixture of growth factors, cytokines, and regulatory molecules that may influence the local wound environment (15–17). Experimental studies have suggested that these components can promote angiogenesis, support keratinocyte and fibroblastic activity, and modulate inflammatory signaling pathways. In immune-mediated dermatoses, local immunomodulatory effects, such as attenuation of pro-inflammatory cytokine signaling, may theoretically contribute to tissue stabilization (6, 16). However, mechanistic effects were not assessed in this study, and such interpretations remain speculative.

### Safety considerations

Safety is a key consideration in pediatric applications. In this series, no treatment-related adverse events were documented. Nevertheless, the limited sample size and follow-up duration preclude definitive conclusions regarding safety, and these findings should be interpreted with caution (17–21).

### Strengths and limitations

Several limitations must be acknowledged. The retrospective design and small sample size limit statistical analysis and increase susceptibility to selection and observational bias. The heterogeneity of diagnoses and concurrent treatments further complicates interpretation. Wound size and percentage reduction were estimated retrospectively from serial photographic documentation using anatomical approximation based on TBSA and regional distribution models. These estimates relied on visual comparison of re-epithelialized versus affected areas rather than direct measurement using calibrated digital tools. Therefore, these parameters represent semi-quantitative measures, reflecting approximate rather than precise or instrument-based quantification.

Standardized wound assessment scales and blinded evaluation were not used, which introduces potential observer bias and limits the objectivity and reproducibility of outcome assessment. Clinical evaluation was performed by the treating team in a non-blinded manner, and no validated scoring system, such as the Pressure Ulcer Scale for Healing or Bates-Jensen tools, or digital planimetric analyses were used. As a result, comparisons across cases and with external studies are inherently limited.

Additionally, detailed molecular characterization of the EV cargo was not available. Although manufacturer-provided data confirmed the presence of vesicle-associated markers (e.g., CD9, CD63, and CD81), comprehensive profiling—including particle size distribution, concentration, purity, and cargo composition (proteins, lipids, and RNA)—was not performed. This lack of standardized EV characterization limits mechanistic interpretation, reproducibility, and alignment with established frameworks such as the MISEV guidelines, which emphasize rigorous and transparent reporting for EV-based studies (22). Taken together, these findings should be considered hypothesis-generating. This case series provides preliminary clinical observations suggesting that MSC-derived secretome-enriched biological dressings may represent a potentially feasible adjunct that warrants further prospective evaluation.

### Future directions

Future prospective studies should incorporate controlled and preferably randomized designs to better delineate the independent contribution of MSC-derived secretome-enriched dressings compared to standard care alone. The use of standardized and validated wound assessment tools (e.g., digital planimetry and validated wound scoring systems) and blinded outcome evaluation would improve objectivity and reproducibility. In addition, detailed physicochemical and molecular characterization of EV—including particle size distribution, concentration (e.g., nanoparticle tracking analysis), cargo profiling (proteomic and RNA content), and purity metrics—should be integrated in accordance with MISEV guidelines to enhance comparability across studies. Correlative analyses exploring biological markers of inflammation, angiogenesis, and tissue repair could further elucidate mechanisms of action. Finally, larger multicenter pediatric cohorts with longer follow-up are needed to better assess safety, durability of response, and potential effects on scarring and functional outcomes.

## Conclusion

In this small retrospective uncontrolled case series, adjunctive topical MSC-derived secretome-enriched biological dressings were used alongside standard care, and progressive re-epithelialization was observed across all cases during follow-up. However, given the observational design, small sample size, heterogeneous diagnoses, and absence of a control group, these findings should be interpreted as descriptive and hypothesis-generating. Despite these limitations, this real-world clinical experience contributes preliminary data in a setting where pediatric evidence remains limited and supports further investigation through well-designed prospective controlled studies incorporating standardized wound assessment methods and detailed EV characterization to better define the therapeutic role and safety profile of these interventions in pediatric regenerative dermatology.

## Data Availability

The datasets presented in this article are not readily available because availability restrictions. Requests to access the datasets should be directed to adrianavalenciaherrera@gmail.com.
